# Pan-membrane pyroptosis of liver induced by gasdermin-encoding mRNAs

**DOI:** 10.1093/nsr/nwaf452

**Published:** 2025-10-21

**Authors:** Yi-Jiao Huang, Lin Li, Guan Yang, Wenlong Ma, Qin Wang, Xiu-Li Yan, Qing Ye, Xing-Yao Huang, Kai Li, Lu Lv, Xiao-Yan Wu, Kaiyan Zhang, Jia Zhou, Xiang Chen, Hui-Sang Lin, Jie Xu, Xue-Tao Zhou, Zhuang-Ran Lin, Xia Zhong, Bo Ying, Yucai Wang, Byung-Ho Kang, Cheng-Feng Qin

**Affiliations:** State Key Laboratory of Pathogen and Biosecurity, Academy of Military Medical Sciences, Beijing 100071, China; State Key Laboratory of Pathogen and Biosecurity, Academy of Military Medical Sciences, Beijing 100071, China; School of Basic Medical Sciences, Tsinghua University, Beijing 100084, China; State Key Laboratory of Medical Proteomics, Beijing Proteome Research Center, National Center for Protein Sciences (Beijing), Beijing Institute of Lifeomics, Beijing 102206, China; School of Life Sciences, Centre for Cell & Developmental Biology and State Key Laboratory of Agrobiotechnology, The Chinese University of Hong Kong, Hong Kong, China; Department of Radiology, The First Affiliated Hospital of University of Science and Technology of China, Division of Life Sciences and Medicine, University of Science and Technology of China, Hefei 230027, China; State Key Laboratory of Pathogen and Biosecurity, Academy of Military Medical Sciences, Beijing 100071, China; State Key Laboratory of Pathogen and Biosecurity, Academy of Military Medical Sciences, Beijing 100071, China; State Key Laboratory of Pathogen and Biosecurity, Academy of Military Medical Sciences, Beijing 100071, China; State Key Laboratory of Pathogen and Biosecurity, Academy of Military Medical Sciences, Beijing 100071, China; State Key Laboratory of Pathogen and Biosecurity, Academy of Military Medical Sciences, Beijing 100071, China; School of Basic Medical Sciences, Tsinghua University, Beijing 100084, China; State Key Laboratory of Pathogen and Biosecurity, Academy of Military Medical Sciences, Beijing 100071, China; School of Life Sciences, Centre for Cell & Developmental Biology and State Key Laboratory of Agrobiotechnology, The Chinese University of Hong Kong, Hong Kong, China; Institute of Chinese Epidemic Disease, Beijing University of Chinese Medicine, Beijing 100029, China; State Key Laboratory of Pathogen and Biosecurity, Academy of Military Medical Sciences, Beijing 100071, China; State Key Laboratory of Medical Proteomics, Beijing Proteome Research Center, National Center for Protein Sciences (Beijing), Beijing Institute of Lifeomics, Beijing 102206, China; State Key Laboratory of Medical Proteomics, Beijing Proteome Research Center, National Center for Protein Sciences (Beijing), Beijing Institute of Lifeomics, Beijing 102206, China; State Key Laboratory of Medical Proteomics, Beijing Proteome Research Center, National Center for Protein Sciences (Beijing), Beijing Institute of Lifeomics, Beijing 102206, China; State Key Laboratory of Medical Proteomics, Beijing Proteome Research Center, National Center for Protein Sciences (Beijing), Beijing Institute of Lifeomics, Beijing 102206, China; Suzhou Abogen Biosciences Co., Ltd., Suzhou 215123, China; Suzhou Abogen Biosciences Co., Ltd., Suzhou 215123, China; Department of Radiology, The First Affiliated Hospital of University of Science and Technology of China, Division of Life Sciences and Medicine, University of Science and Technology of China, Hefei 230027, China; School of Life Sciences, Centre for Cell & Developmental Biology and State Key Laboratory of Agrobiotechnology, The Chinese University of Hong Kong, Hong Kong, China; State Key Laboratory of Pathogen and Biosecurity, Academy of Military Medical Sciences, Beijing 100071, China; Research Unit of Discovery and Tracing of Natural Focus Diseases, Chinese Academy of Medical Sciences, Beijing 100071, China

**Keywords:** pyroptosis, GSDMD, liver damage, mRNA-LNP, non-human primates

## Abstract

Gasdermin (GSDM)-mediated pyroptosis has been extensively visualized *in vitro* and linked to multiple physiological and pathological processes. However, the *in vivo* phenotype and clinical outcome of pyroptosis remain undetermined. Here, we sought to profile *in vivo* pyroptosis using lipid-nanoparticle (LNP)-encapsulated mRNA encoding the pore-forming N-terminal of GSDMD (GSDMD^NT^). Upon intravenous (IV) injection, robust expression of GSDMD^NT^ led to acute liver damage, systemic inflammation and sudden death, both in mice and non-human primates, which could be reversed by the GSDMD inhibitor disulfiram or glucocorticoids. Imaging techniques revealed that GSDMD^NT^ targeted both plasma membrane and intracellular membranous organelles, causing membrane rupture and organelle swelling, as well as the formation of intracellular membranous vacuoles. Furthermore, heterologous expression of other GSDM members also caused pyroptosis, both *in vitro* and *in vivo*, with varying magnitude. These findings provide insights into the dynamic characteristics of pyroptotic organ injury-related diseases and offer the basis for developing GSDM-based therapeutics.

## INTRODUCTION

Pyroptosis is a rapid form of cell death mediated by the N-terminal (NT) of gasdermins (GSDMs), which was initially considered to induce plasma membrane perforation and formation of large outwardly ballooning bubbles [1–6]. Pores in the plasma membrane lead to the release of cellular alarmins, such as adenosine triphosphate (ATP), cleaved GSDMs and cytokines, into the extracellular environment, thereby amplifying immune response *in vivo* [7–9]. This feature makes GSDM-mediated pyroptosis attract attention to anti-tumor therapy. Activation or delivery of GSDMs *in vivo* can induce pyroptosis in tumor cells and enhance anti-tumor immunity [7,10–14]. GSDME and GSDMB have been the earliest reported to activate pyroptosis in tumor cells for potential therapeutic effects [12,15]. Recently, a small-molecule, 6,7-dichloro-2-methylsulfonyl-3-*N*-tert-butylaminoquinoxaline (DMB), was identified as a potent GSDMD agonist [13]. DMB induces GSDMD-dependent pyroptosis in tumor cells and enhances anti-tumor immunity. However, the levels of endogenous expression of GSDMs vary in cell types and states, making the treatment complicated.

Compared to endogenous activation of GSDMs, exogenously delivering activated GSDMs into cells may result in faster and more pronounced pyroptotic effects, as well as making them more dose-controllable. Nanoparticles are fast-developing carriers for effective delivery of nucleic acids or proteins, most widely used for vaccines and gradually being used for tumor therapy [16,17]. First, the nanoparticle-conjugated GSDMA protein was delivered into tumor-bearing mice in conjunction with a bioorthogonal chemical system, highlighting the potential of GSDM-based nanoparticle therapy for tumors [10]. Following that, as the widespread application of lipid-nanoparticles (LNPs), especially for SARS-CoV-2 immunization [18–22], LNP-encapsulated *GSDMB* mRNA and *GSDMD* circular RNA (circRNA) were used for tumor treatment in mice models, triggering both the pyroptosis of tumor cells and anti-tumor immunity [22,23].

While the strong pyroptosis induced by GSDMs makes the killing of tumors robust, it may be associated with severe side effects on normal tissues as well. The liver is the major site of drug metabolism, contributing to approximately 70% of drug elimination in humans [24], and functions as the primary target organ for LNP delivery [25,26]. It is unavoidable to affect the liver, leading to drug-induced liver injury (DILI) [27], when administered with GSDM activators or GSDM-related nanoparticles. Therefore, characterizing pyroptosis in liver is conducive to determining the level of pyroptosis *in vivo* for further evaluating the safety and efficacy.

Previous studies on pyroptosis have primarily focused on monolayer or free-cell-state cultures such as cell lines or immunocytes isolated from the body. *In vitro*, GSDM-induced pyroptosis is characterized by the formation of large ballooning bubbles in the damaged membrane and the flattening of dying cells [2,7]. However, in the authentic *in vivo* milieu, solid tissues and organs are intimately conjoined, which may impede the occurrence of typical outward bubbling phenomena observed at the cellular level. For example, the liver’s 3D architecture with comprehensive intercellular junctions provides mutual support and stability [28,29]. This structural feature will result in distinct morphological changes during pyroptotic liver damage compared to pyroptosis observed *in vitro*. Moreover, unlike relative static culture *in vitro*, the flowing bloodstream makes the *in vivo* microenvironment more complicated.

In this study, by using emerging LNP-encapsulated mRNA technology, we successfully induced *in vivo* pyroptosis in the liver of mice and non-human primates, and investigated extensive intravital pathological changes and unexpected systemic responses, establishing critical characteristics for pyroptosis in the solid organ.

## RESULTS

### GSDM-based therapy caused liver-specific pathological changes in mice

**Figure 1.**
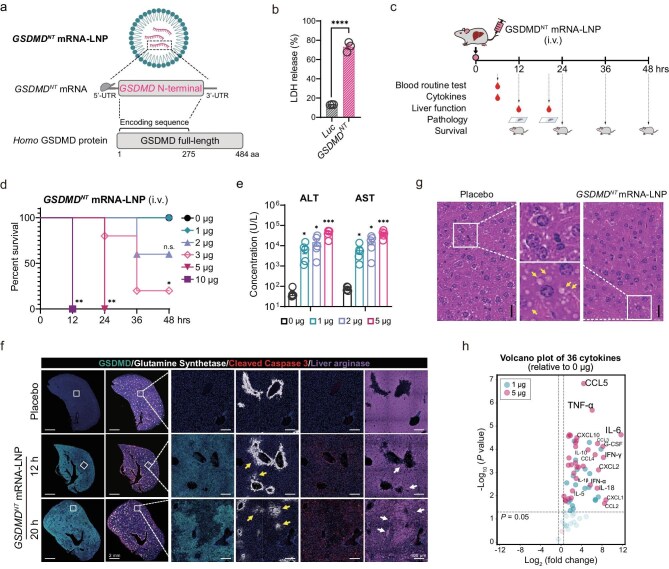
*GSDMD^NT^* mRNA-LNP injection led to mortality in mice accompanied by impaired liver function and elevated serum cytokines. (a) Schematic diagram of *GSDMD^NT^* mRNA-LNP design. LNP-encapsulated *GSDMD^NT^* mRNA encoded the N-terminal domain of *Homo* GSDMD. (b) LDH-release-based cell death of HEK293T cells transfected with *GSDMD^NT^* mRNA for 6 h. (c) Schematic diagram of *GSDMD^NT^* mRNA-LNP delivery and detection in mice. (d and e) Survival curves (d) and liver function parameters (e) of mice administered varying IV doses of *GSDMD^NT^* mRNA-LNP (*n* = 5). The concentrations of serum ALT or AST were measured 20 hpi (e). Statistical analysis of the survival was performed with a log-rank (Mantel–Cox) test (d). (f) Immunofluorescent analysis of mouse livers after administration of 5 μg of *GSDMD^NT^* mRNA-LNP or placebo (IV) for 12 or 20 h. Overexpression of GSDMD^NT^ stained green. Yellow or white arrows indicate damage to the area adjacent to the hepatic vein or disruption in the uniformity of hepatocyte distribution. Magnifications of the boxed areas are shown on the right, respectively. (g) H&E staining of mice livers after administration of 5 μg of *GSDMD^NT^* mRNA-LNP or placebo (IV) for 20 h. Yellow arrows indicate intracellular vacuoles. Boxed areas are shown in the middle at higher magnifications. Scale bar, 20 μm. (h) Serum cytokine levels of mice injected IV with the indicated doses of *GSDMD^NT^* mRNA-LNP for 6 h (*n* = 3). The volcano plot shows the fold changes (*x*-axis) and *P* values (*y*-axis) of 36 cytokines detected by Luminex xMAP technology, relative to the 0 μg group; vertical dashed lines indicate a 1.5-fold increase or decrease; light-colored transparent circles represent cytokines with no statistically significant difference. Data (b and e) are shown as mean ± SEM, unpaired *t*-test. (n.s., not significant; * *P* < 0.05; ** *P* < 0.01; *** *P* < 0.001; **** *P* < 0.0001).

LNPs are widely applied to deliver mRNA *in vivo* for vaccination, and are gradually being applied in tumor therapy [16,18,20,21]. Initially, we sought to treat tumors by using GSDM-encoding mRNAs. Considering GSDMD as the best-characterized member of GSDM proteins [30], we chose the NT [amino acid (aa) 1–275] of human GSDMD (GSDMD^NT^, the activated form of GSDMD) [1] for further investigation (Fig. 1a). Subsequent transfection of cells with GSDMD^NT^-encoding mRNA resulted in evident pyroptosis induction (Fig. 1b; Fig. S1a). The mRNA-LNP formulations were prepared and characterized (Fig. S1b) as described previously for subsequent *in vivo* experiments [31]. Briefly, *GSDMD^NT^* mRNA-LNP was administered intratumorally (IT) in the B16-F10 mouse melanoma model, which is widely used for tumor studies [32] (Fig. S1c). As shown in Fig. S1d–f, treatment with *GSDMD^NT^* mRNA-LNP significantly reduced both tumor volume and mass compared to placebo controls. However, although the treated group did not exhibit significantly impaired liver function (Fig. S1g), the TdT-mediated dUTP nick-end labeling (TUNEL) assay and hematoxylin and eosin (H&E) staining of liver from the treated mice showed noticeable hepatocellular cell death (Fig. S1h) and dispersed vacuoles within hepatic cells (Fig. S1i). These unusual observations indicate potential liver damage and specific pathological changes associated with GSDM-based therapies.

### *GSDMD^NT^* mRNA-LNP injection led to mortality in mice, accompanied by impaired liver function and elevated serum cytokines

We then aimed to investigate the direct impact of *GSDMD^NT^* mRNA-LNP injection on mouse liver. Mice were injected IV with varying doses of *GSDMD^NT^* mRNA-LNP, followed by relevant assessments including clinical manifestations, survival rates, liver function parameters, blood routine tests and serum cytokine levels etc. (Fig. 1c). Strikingly, *GSDMD^NT^* mRNA-LNP injection led to dose-dependent mortality; mice receiving 5 or 10 μg of *GSDMD^NT^* mRNA-LNP exhibited reduced locomotor activity, decreased food consumption and lowered body temperature (data not shown), ultimately succumbing within 24 or 12 h, respectively (Fig. 1d). Even at a low dose of 2 μg, *GSDMD^NT^* mRNA-LNP caused instances of sudden death within 36 h post-injection (hpi). Although complete blood count did not reveal any significant changes induced by *GSDMD^NT^* mRNA-LNP at 20 hpi (Fig. S2a), analysis of blood chemistry panels showed that *GSDMD^NT^* mRNA-LNP led to a significant increase in aspartate aminotransferase (AST) and alanine transaminase (ALT) in a dose-dependent manner (Fig. 1e), indicating rapid impairment of liver function. Consistently, multiple staining of liver sections revealed robust expression of GSDMD resulting in disruption of hepatic venous zones (indicated by glutamine synthetase predominantly localized to the hepatic venous outflow region [33]), uneven distribution of hepatocytes (indicated by liver arginase expressed in the cytoplasm of hepatocytes [34]) and extensive cell death marked by caspase-3 staining (Fig. 1f).

Liver pathology was next assessed through immunohistochemistry (IHC). As shown in Fig. 1g, hepatic cells exhibited an unusually abundant distribution of dispersed vacuoles, which were larger in size compared to those induced during tumor treatment, as shown in Fig. S1i. Oil red O staining demonstrated that these vacuoles differed from lipid droplets, although a certain amount of lipid content could be detected within them (Fig. S2b). This observation implies that the presence of these intracellular vacuoles serves as a typical pathological characteristic associated with GSDMD-mediated pyroptosis in liver.

Furthermore, we investigated the systemic immune responses following *GSDMD^NT^* mRNA-LNP treatment. Due to the short half-lives of serum cytokines, mouse sera were collected at the early timepoint of 6 hpi for the comprehensive detection of cytokines. As shown in Fig. 1h and Fig. S2c, a significant and dose-dependent upregulation of a wide array of serum cytokines was observed, including interleukins (IL-1α, IL-6, IL-18), interferons (IFN-α, IFN-γ), tumor necrosis factor alpha (TNF-α) and colony-stimulating factors (G-CSF, GM-CSF), as well as chemokines belonging to the CC subfamily (CCL2, CCL5) or CXC subfamily (CXCL1, CXCL10). Many of these cytokines have been previously reported to be associated with liver damage [35–38]. Meanwhile, enhanced expression of IL-1, IL-18, and NF-κB in the liver was consistently observed at both 12 and 20 hpi (Fig. S2d). These results demonstrated that heterologous GSDMD^NT^ expression in liver caused sudden death associated with acute liver damage and systemic inflammation.

### GSDMD abolished intracellular adhesion in liver

**Figure 2.**
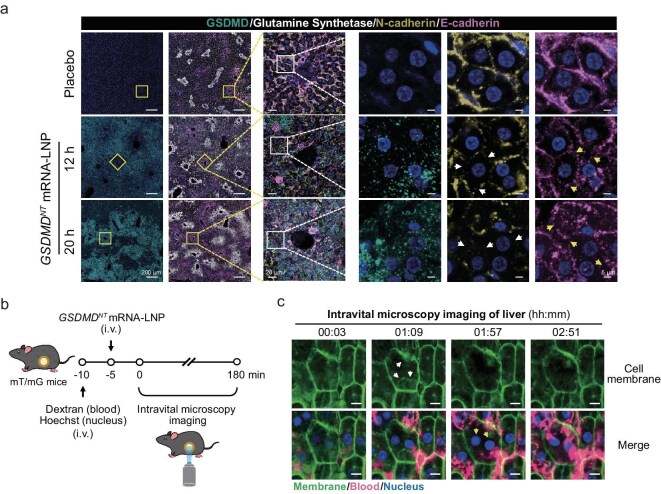
GSDMD^NT^ abolished intracellular adhesion in liver. (a) Immunofluorescent analysis of mouse livers after administration of 5 μg of *GSDMD^NT^* mRNA-LNP or placebo (IV) for 12 or 20 h. Overexpression of GSDMD^NT^ stained green. Hepatocytes surrounding the hepatic venules stained white. Magnifications of the boxed areas are shown on the right, respectively. Arrows indicate blurred intercellular adhesion between hepatocytes. (b) Schematic diagram of abdominal window model and intravital microscopy imaging of mouse hepatic cells after *GSDMD^NT^* mRNA-LNP administration. mT/mG mice with fluorescent cell membrane underwent surgical creation of an abdominal liver window. Mice were pre-treated with FITC-dextran and Hoechst for 10 min, followed by IV injection of 5 μg of *GSDMD^NT^* mRNA-LNP for 5 min. Blood vessels and hepatic cell nuclei were visualized for over 180 min with a confocal microscope. (c) Intravital real-time cell imaging was performed by a confocal microscope at 3-min intervals. Representative images are shown at the indicated timepoints. Green indicates cell membrane; FITC-dextran (false cherry) stained the blood; Hoechst (blue) stained the nuclei. White arrows show membranes separated from their original framework and concaved inward. Yellow arrows show karyopyknosis. Scale bar, 10 μm.

To examine the fine structural alterations of the liver, we employed E- and N-cadherins to visualize distinct cell boundaries [39,40], and observed that increasing expression of GSDMD^NT^ in liver led to a blurring of clear intercellular adhesions between hepatocytes and hepatic sinusoids (Fig. 2a); by 20 hpi, these intercellular connections were almost completely eradicated. These findings reflect severe structural damage inflicted on the liver by GSDMD^NT^.

We then sought to characterize the real-time dynamics of liver cell membranes upon *GSDMD^NT^* mRNA-LNP injection by using the newly developed mT/mG mouse model expressing a two-color fluorescence specifically localized to the cell membrane [41]. Intravital microscopy imaging of the liver was performed for 3 hpi of fluorescein isothiocyanate–dextran (FITC-dextran) (to visualize blood fluid with false cherry fluorescence [17]) and Hoechst solution (for nuclear staining), along with *GSDMD^NT^* mRNA-LNP treatment (Fig. 2b). As shown in Fig. 2c and Movie S1, after 1 h of monitoring (timepoint of 01:09), certain cell membranes separated from the original framework, concaved inward, and subsequently the inward bubbles disappeared within 1 h (timepoint of 01:57), presenting different morphological changes compared to the vacuoles observed in Fig. 1g. Given that the liver possesses a rich blood supply, with an average portal venous pressure of 10 mmHg [42], the ruptured membranes offered opportunities for immediate bloodstream infiltration, leading to cellular filling and karyopyknosis (timepoint of 01:57). However, due to the tight intercellular connections of hepatocytes, they did not collapse or flatten during this process (timepoint of 02:51). These results indicate that GSDMD^NT^-induced morphological changes of cell membranes of *in vivo* pyroptosis were distinct from *in vitro* pyroptosis. Nonetheless, as observed under a wider field of view in Movie S1, it was noted that in numerous cells, karyopyknosis occurred prior to changes in membrane integrity or blood infiltration, indicating potential effects associated with the characteristic intracellular vacuoles.

By integrating these results with the changes of liver structure and hepatic cells shown in Fig. 1f and g, we have observed the dynamic characterization and morphological changes of cell membranes associated with GSDMD-mediated *in vivo* pyroptosis, and described the pathological characteristics of pyroptotic liver damage. GSDMD^NT^ overexpression caused cell membrane disruption, intracellular vacuole formation and blood infiltration or karyopyknosis, consequently leading to the disruption of the hepatic structural network.

### GSDMD^NT^ non-selectively targeted membranous organelles

**Figure 3.**
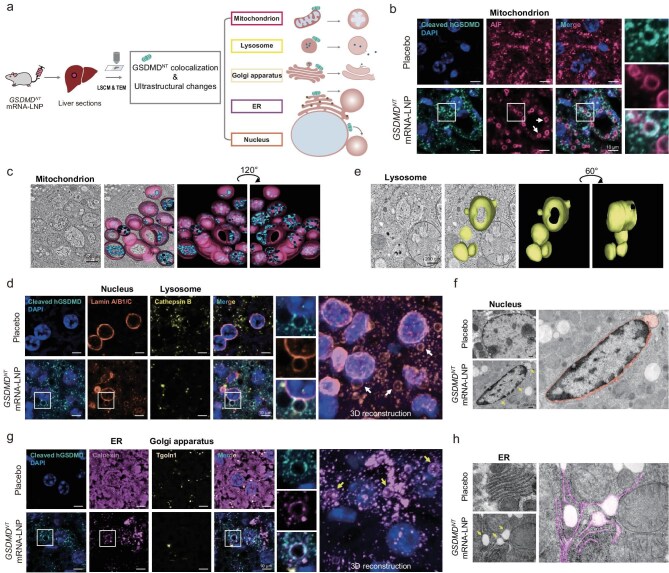
GSDMD^NT^ non-selectively targeted membranous organelles. (a) Schematic diagram of confocal and electron microscopy imaging of mouse hepatocytes after *GSDMD^NT^* mRNA-LNP treatment. Membranes of intracellular organelles are colored by different fluorescence matching with the color of textboxes. GSDMD^NT^, green (cleaved hGSDMD); mitochondrial membranes, cherry (AIF); lysosomal membranes, yellow (cathepsin B); nuclear envelopes, orange (lamin A/B1/C); ER membranes, purple (calnexin). (b, d and g) Immunofluorescent analysis of mouse hepatocytes showing mitochondria (b), lysosomes and nuclei (d) or ER (g). The mice were IV injected with 5 μg of *GSDMD^NT^* mRNA-LNP or placebo for 3 h (b) or 20 h (d and g). Boxed areas show GSDMD^NT^ colocalized with organelles. In (d) and (g), 3D reconstruction of nuclear envelopes (d) or ER membranes (g) is shown on the rightmost panel. Nuclei are stained blue (DAPI). Scale bar, 10 μm. (c, e, f and h) Representative TEM images of swollen mitochondria (c), lysosomes (e), nuclei (f) or ER (h). The mice were IV injected with 5 μg of *GSDMD^NT^* mRNA-LNP or placebo for 12 h. In (c) and (e), 3D reconstruction tomograms (right) are shown; mitochondrial outer membranes are highlighted in cherry and the damaged ridge inside mitochondria are presented in cyan (c); lysosomal outer membranes are highlighted in yellow (e). In (f) and (h), the swollen nuclear envelope is marked in orange (f); the swollen ER is marked in purple (h); arrows indicate swollen or damaged organelles. Scale bar, 500 nm (c and f), 200 nm (e and h).

To investigate the mechanism by which GSDMD^NT^ induced cell death without plasma membrane rupture and its association with characteristic vacuoles, we systematically studied subcellular organelles in hepatocytes using laser scanning confocal microscopy (LSCM) and transmission electron microscopy (TEM) (Fig. 3a). Previous studies have reported that in addition to targeting the plasma membrane, the NT of GSDMs can also target mitochondria in some immune cells [43,44]. To specifically identify mitochondria, we employed apoptosis-inducing factor (AIF) as a marker localized within the intermembrane space. As Fig. 3b shows, GSDMD^NT^ colocalized with mitochondria and damaged them, leading to mitochondrial swelling (cherry-colored circles) and even disappeared. TEM analysis further revealed swollen mitochondria with indistinct cristae and vacuolar alterations caused by pore formation induced by GSDMD^NT^ (Fig. 3c; Fig. S3a).

More importantly, we observed significant morphological changes in various membranous organelles, including lysosomes, endoplasmic reticulum (ER), Golgi apparatus and the nucleus (Fig. 3d–h; Fig. S3b and c). Lysosomes function as cellular stomachs filled with numerous hydrolases capable of digesting most cellular macromolecules [45]. Extensive lysosomal membrane permeabilization (LMP) leading to the release of contents can result in uncontrolled necrosis [46]. We observed that GSDMD^NT^ induced early-stage lysosome (stained by cathepsin B) swelling (Fig. S3b and  c), which gradually dissipated over time (Fig. 3d). Interestingly, by 3D TEM reconstruction, we visualized the ultrastructure of a swelling and leaking lysosome (Fig. 3e; Movie S2). These observations indicate the presence of LMP and its associated cell death in the *in vivo* pyroptosis.

As the largest organelle in the cell, the ER consists of the nuclear envelope and the peripheral ER, composed of interconnected tubules and sheets called cisternae [47,48]. Upon GSDMD^NT^ treatment, the nuclear envelope was disrupted, along with alterations in nuclear integrity (stained with lamin A/B1/C), including nuclear shrinking, envelope rupture and even bubble formation that may correspond to the characteristic vacuoles (Fig. 3d and f; Movie S3). The peripheral ER was labeled with calnexin. Compared to the evenly distributed and abundant ER observed in normal hepatocytes, GSDMD^NT^-treated cells exhibited thinly scattered ER, which also formed bubbles colocalized with GSDMD^NT^ (Fig. 3g and h). Interestingly, during nuclear envelope or ER bubble formation events, it became difficult to observe lysosomes or Golgi apparatus (stained by TgoIn1) within the same cell. The reason might be that cytoplasmic organelles and granules typically occupied the hepatocyte cytoplasm, hindering early detection of pyroptosis-induced changes; however, once these organelles were disrupted or collapsed, space was made to allow ballooning of interconnected ER lumens or peeling of ruptured nuclear envelope, leading to conspicuous vacuole formation. These results suggest that swollen mitochondria, isolated nuclear envelope or damaged ER may constitute the characteristic vacuoles observed in Fig. 1g. Furthermore, these abnormalities could potentially lead to subsequent destructive effects such as early karyopyknosis observed in Movie S1.

In conjunction with the above findings on morphological alterations of *in vivo* pyroptosis, our study demonstrates that GSDMD^NT^ non-specifically targets cellular membranes and induces damage to various membranous organelles, ultimately leading to extensive cell death.

### *GSDMD^NT^* mRNA-LNP causes liver pyroptosis and sudden death in non-human primates

**Figure 4.**
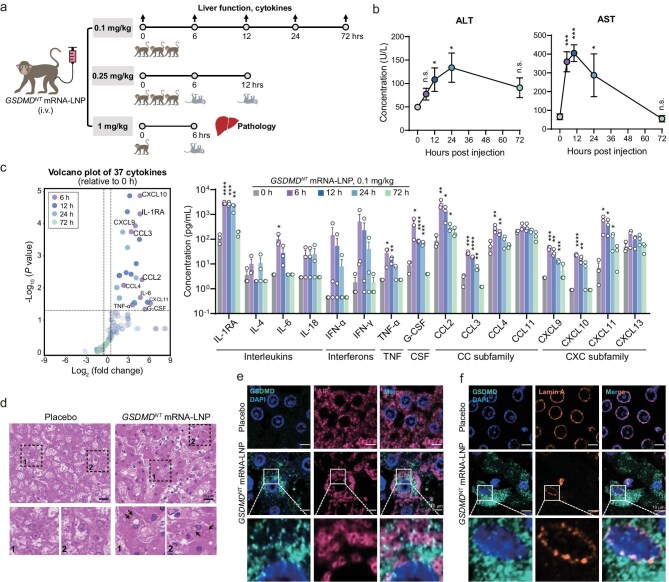
*GSDMD^NT^* mRNA-LNP causes liver pyroptosis and sudden death in non-human primates. (a) Schematic diagram of the experiment in cynomolgus macaques administered IV *GSDMD^NT^* mRNA-LNP at doses of 0.1 (*n* = 3), 0.25 (*n* = 3) or 1 (*n* = 1) mg/kg. (b and c) Liver function results (b) and cytokine results (c) of monkeys administered *GSDMD^NT^* mRNA-LNP at a dose of 0.1 mg/kg via IV drip. Blood was collected at 0, 6, 12, 24 or 72 h post- administration. The line chart (b) shows the changes in serum ALT or AST (U/L) level over time after treatment. In (c), the volcano plot shows the fold changes (*x*-axis) and *P* values (*y*-axis) of 37 cytokines detected by Luminex xMAP technology, relative to 0 h; vertical dashed lines indicate a 1.5-fold increase or decrease; light-colored transparent circles represent cytokines with no statistically significant difference; the histogram displays concentrations of partial cytokines. Data are shown as mean ± SEM, unpaired *t*-test. (n.s., not significant; * *P* < 0.05; ** *P* < 0.01; *** *P* < 0.001; **** *P* < 0.0001). (d) H&E staining of the liver from monkeys treated with 1 mg/kg *GSDMD^NT^* mRNA-LNP or placebo for approximately 6 h. Boxed areas in each image are shown below at higher magnifications, showing dispersed vacuoles in hepatic cells of the GSDMD^NT^ group. (e and f) Immunofluorescent analysis of monkey hepatocytes, showing mitochondria (e) or nuclear envelopes (f). GSDMD^NT^ is stained green (cleaved hGSDMD); nuclei are stained blue (DAPI); mitochondrial membranes are stained cherry (AIF); nuclear envelopes are stained orange (lamin A).

Considering the closer resemblance of liver metabolism between humans and non-human primates compared to mice, we further expand our findings to non-human primates. Cynomolgus macaques were treated IV with different doses of *GSDMD^NT^* mRNA-LNP, and serum biochemical assays and pathology were monitored (Fig. 4a). As shown in Fig. 4b and c, and Fig. S4a, a lower dose (0.1 mg/kg) of *GSDMD^NT^* mRNA-LNP administration temporarily impaired liver function (Fig. 4b) and increased levels of multiple serum cytokines (Fig. 4c), and all animals survived without any obvious clinical features. Moreover, a higher dosage at 0.25 or 1 mg/kg resulted in higher mortalities (66.7% or 100%, respectively); the death occurred within a 12-h or even 6-h period (Fig. 4a; Fig. S4a).

To validate whether *in vivo* pyroptosis in the liver observed in mice also exists in monkeys, we collected tissue samples after 6 h when the monkey administered a high dose of 1 mg/kg was approaching the terminal stage. H&E staining and multiplex immunofluorescence staining of liver sections showed the formation of characteristic vacuoles in monkey hepatocytes, destruction of the hepatic structural network and upregulation of inflammatory factors in the liver (Fig. 4d; Fig. S4b). More importantly, distinct mitochondrial swelling and nuclear envelope rupture were detected, along with colocalized GSDMD^NT^ (Fig. 4e and f). These consistent results from both mice and non-human primates collectively profiled the unique characterization of GSDMD-induced pyroptosis in solid organs, as well as highlighting the severe clinical outcome linked to GSDM-based therapies.

### GSDMD^NT^-induced mortality could be attenuated by the GSDMD inhibitor disulfiram or corticosteroids in mice

**Figure 5.**
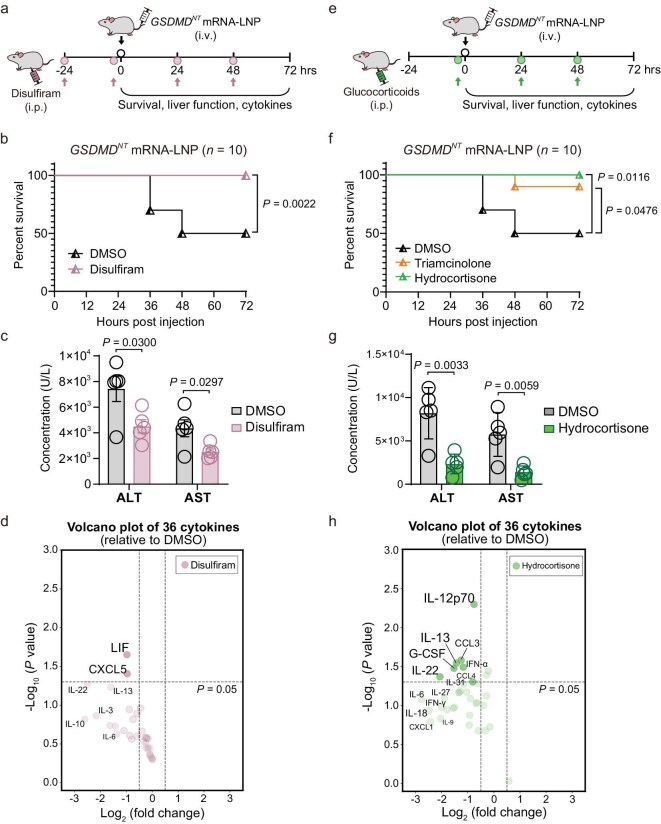
GSDMD^NT^-induced mortality could be attenuated by a GSDMD inhibitor or glucocorticoids in mouse. (a and e) Schematic diagram of disulfiram (a) or glucocorticoid (e) treatment for GSDMD^NT^-injected mice. Pink (a) or green (e) arrows indicate the timepoints of inhibitor administration. (b and f) Survival curves of *GSDMD*^NT^ mRNA-LNP-injected mice. The GSDMD^NT^-injected mice (*n* = 10) were administered DMSO or disulfiram (50 mg/kg) (b), and triamcinolone (5 mg/kg) or hydrocortisone (5 mg/kg) intraperitoneally (IP) (f). Statistical analysis was performed with a log-rank (Mantel–Cox) test. (c and g) Serum levels of ALT and AST in GSDMD^NT^-injected mice (*n* = 5) with IP administered DMSO, disulfiram (c) or hydrocortisone (g). Data are shown as mean ± SEM, unpaired *t*-test. (d and h) Volcano plots of serum cytokine levels in GSDMD^NT^-injected mice (*n* = 3) with IP administered disulfiram (d) or hydrocortisone (h). *x*-axis indicates the fold changes of 36 cytokines relative to the DMSO group; *y*-axis shows *P* values; vertical dashed lines indicate a 1.5-fold increase or decrease; light-colored transparent circles represent cytokines with no statistically significant difference. Data are shown as mean, unpaired *t*-test.

To further elucidate whether mouse mortality induced by GSDMD^NT^ could be rescued, subsequent animal experiments were conducted using disulfiram, a well-identified GSDMD inhibitor [49]. As expected, disulfiram not only inhibited *GSDMD^NT^* mRNA-induced pyroptosis in HEK293T (Fig. S5a), but its administration also efficiently reduced mouse mortality caused by *GSDMD^NT^* mRNA-LNP (Fig. 5a and b), along with significantly reduced serum ALT and AST levels (Fig. 5c). Meanwhile, the early elevated systemic inflammatory response triggered by *GSDMD^NT^* mRNA-LNP was slightly weakened following disulfiram treatment (Fig. 5d). Moreover, we constructed a GSDMD^NT^ mutant containing a mutated β1–β2 loop that lacks pore-forming capability and pyroptotic capabilities [50] (Fig. S5b). As shown in Fig. S5c–e, this mutant had no effect on survival or liver function. These data suggest that GSDMD activity is the primary determinant of the observed acute liver failure and sudden death in mice.

In addition to the direct hepatocyte damage caused by GSDMD^NT^, the subsequent amplification of this damage mediated by high levels of cytokines or other danger molecules can also lead to further systemic injuries, a phenomenon known as a cytokine storm [51,52]. Corticosteroids are widely employed in the clinical treatment of various autoimmune and inflammatory disorders, as well as liver failure, due to their potent anti-inflammatory and immunosuppressive properties [53–55]. We administered corticosteroids, triamcinolone or hydrocortisone to GSDMD^NT^-treated mice (Fig. 5e). As expected, both drugs attenuated mouse mortality (Fig. 5f), along with significantly reduced serum ALT and AST levels (Fig. 5g) and a significant decrease in serum levels of multiple cytokines like interleukins and the chemokine CC subfamily (Fig. 5h). However, when *GSDMD^NT^* mRNA-LNP was administered to severely immunodeficient B-NDG mice, which lack mature T cells, B cells and functional NK cells and exhibit defects in cytokine signal transduction, mortality remained unchanged, despite significantly reduced levels of serum cytokines (Fig. S5f and g). This suggests that corticosteroids alleviated mortality primarily by attenuating liver damage. These results support the conclusion that liver damage was predominantly induced by GSDMD^NT^-mediated *in vivo* pyroptosis rather than a generalized systemic inflammatory response.

### GSDMB, but not GSDMA, GSDMC or GSDME, also causes obvious pyroptosis in mouse liver

**Figure 6.**
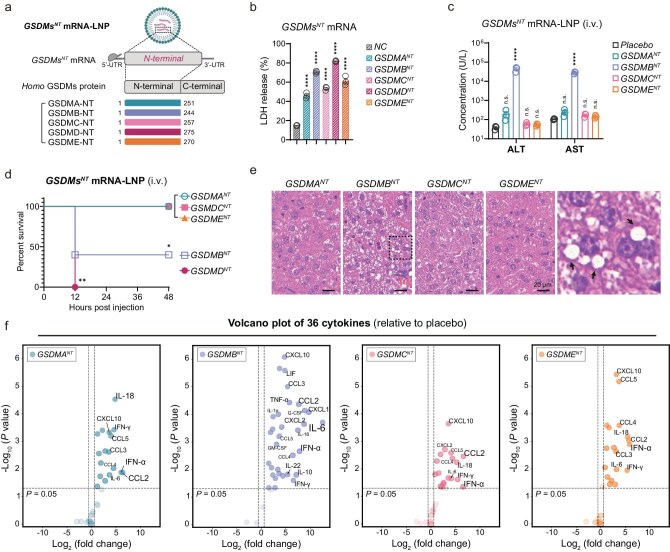
GSDMB, but not GSDMA, GSDMC or GSDME, also caused obvious pyroptosis in liver. (a) Schematic diagram of *GSDM^NT^* mRNA-LNP design. *GSDM^NT^* mRNA encoded the N-terminal domain of *Homo* GSDMs. (b) LDH-release-based cell death of HEK293T cells transfected with different *GSDM^NT^* mRNAs for 6 h. (c and d) Survival curves (*n* = 5) and liver function parameters (*n* = 3) of mice administrated 10 μg of different IV *GSDM^NT^* mRNA-LNPs. Serum ALT and AST concentrations were measured 20 hpi (c). Statistical analysis of survival was performed with a log-rank (Mantel–Cox) test (d). (e) H&E staining of mouse livers after administration of 10 μg of *GSDM*^NT^ mRNA-LNPs for 20 h. Boxed areas in *GSDMB^NT^* group are shown at higher magnifications. (f) Serum cytokine levels of mice injected IV with 10 μg of different *GSDM^NT^* mRNA-LNPs for 6 h (*n* = 3). The volcano plot shows the fold changes (*x*-axis) and *P* values (*y*-axis) of 36 cytokines detected by Luminex xMAP technology, relative to the placebo group. Vertical dashed lines indicate a 1.5-fold increase or decrease; light-colored transparent circles represent cytokines with no statistically significant difference. Data in (b, c and f) are shown as mean ± SEM, unpaired *t*-test. (n.s., not significant; * *P* < 0.05; ** *P* < 0.01; *** *P* < 0.001; **** *P* < 0.0001).

The human GSDM family consists of six members: GSDMA, GSDMB, GSDMC, GSDMD, GSDME and DFNB59 [7]. All the members, except for DFNB59, can undergo cleavage and activation similar to that of GSDMD for membrane perforation [56]. To assess their ability in inducing pyroptosis, we generated truncated mRNA constructs for *GSDMA, GSDMB, GSDMC* and *GSDME* and evaluated pyroptotic damage both *in vitro* and *in vivo* (Fig. 6a). As expected, all *GSDM^NT^* mRNAs exhibited significant *in vitro* lethality and robust expression, as evidenced by the addition of a FLAG tag downstream of GSDM^NT^ (Fig. 6b; Fig. S6a). Subsequently, *GSDM^NT^* mRNA-LNP was administered to mice, and *in vivo* pyroptosis-related test parameters were assessed. Interestingly, except for GSDMD, only GSDMB impaired liver function and led to mortality among mice (Fig. 6c and d). Pathologically, GSDMB induced characteristic vacuoles (Fig. 6e) and a noticeable elevation in inflammatory responses (Fig. 6f), while the same dose of GSDMA, GSDMC or GSDME failed to cause any clinical syndromes or significant pathological changes. These findings highlighted the complexity of the *in vivo* impact of pyroptosis caused by different GSDM members.

## DISCUSSION

In this study, we provide a comprehensive characterization of *in vivo* pyroptosis induced by *GSDMD^NT^* mRNA-LNP, revealing its profound impact on liver function and systemic inflammation in both murine and non-human primate models. Our findings demonstrate that even low doses of *GSDMD^NT^* mRNA-LNP trigger severe pyroptosis in the liver, leading to acute hepatic failure, cytokine storms and rapid mortality. Importantly, these effects were reversible through pharmacological interventions, including the GSDMD inhibitor disulfiram and anti-inflammatory corticosteroids. Pathologically, we identified intracellular vacuoles as a hallmark of GSDMD-induced organelle damage, a phenomenon we term ‘pan-membrane pyroptosis’. This concept expands our understanding of pyroptosis beyond plasma membrane perforation, encompassing non-selective disruption of mitochondrial, lysosomal, ER and nuclear membranes. Furthermore, we uncovered functional divergence among GSDM family members, with only GSDMD and GSDMB exhibiting significant hepatotoxicity *in vivo*.

Traditionally, GSDMs were thought to primarily target the plasma membrane, forming pores that release pro-inflammatory cytokines and induce lytic cell death. However, recent studies have hinted at broader organelle involvement, particularly in immune cells [30,43,44,57]. Our work establishes that GSDMD indiscriminately disrupts all membrane-bound organelles, from mitochondria to the nuclear envelope, leading to a spectrum of cell death phenotypes. This is also applicable to *in vitro* pyroptosis. As shown in Fig. S3d and Movie S4, the lysosome-marked HEK293T cell line was transfected with *GSDMD^NT^* mRNA; within 3 h, lysosomes ruptured and cells died like in apoptosis, which was characterized by nucleus shrinkage and cell rounding; however, the typical characteristic of *in vitro* pyroptosis, large bubble formation, was not observed until after 7 h. These results suggest that organelle damage may dictate the kinetics and morphology of pyroptosis.

The therapeutic potential of GSDMs, particularly in cancer immunotherapy, has garnered significant interest [10,12–15]. However, our findings underscore the risks of systemic GSDM activation, as even localized delivery (e.g. intratumoral) can spill over to damage critical organs like the liver. Notably, GSDMB emerged as the only family member besides GSDMD capable of inducing hepatotoxicity, highlighting functional divergence that could inform safer therapeutic design. For example, GSDMA, GSDMC and GSDME, which showed minimal liver toxicity, may be preferable for immune activation strategies.

Beyond pyroptosis, GSDMD has been implicated in ‘hyperactivation’, where cells secrete cytokines without undergoing lysis [7,58,59]. Our data suggest a mechanistic basis for this phenomenon: GSDMD may initially target proximal organelles (e.g. mitochondria, ER) before reaching the plasma membrane. If organelle damage is limited or rapidly repaired (e.g. nuclear envelope resealing [60]), cells may survive while releasing inflammatory mediators. It is noteworthy that hepatocytes have been implicated in pyroptosis across various diseases [61–63], yet they resist overt pyroptotic cell death even following *in vivo* adenoviral delivery of active caspase-11, suggesting a state of cellular hyperactivation [64]. This model also aligns with observations in some viral infections, such as Zika virus, where low-level GSDMD activation by viral proteases triggers cytokine release without overt cell death [65,66]. Future studies should explore whether organelle-specific GSDMD targeting can be harnessed to fine-tune immune responses.

The rapid progression of GSDM-based therapies, particularly in oncology [13,23,67], necessitates rigorous safety evaluations. Our work highlights the liver as a critical vulnerability, emphasizing the need for organ-specific delivery systems or combination therapies to mitigate off-target effects. The efficacy of disulfiram and corticosteroids in rescuing GSDMD-induced toxicity provides a proof of concept for pharmacological modulation of pyroptosis, offering potential strategies to manage treatment-related adverse events.

While our study provides a detailed mechanistic framework, several questions remain. First, the molecular basis for GSDMD’s non-selective membrane targeting is unclear. Second, the functional divergence among GSDM family members warrants further investigation, particularly in the context of tissue-specific expression and activation. Finally, the long-term consequences of sublethal GSDMD activation (e.g. hyperactivation) on organ function and immune homeostasis remain unexplored.

By elucidating the mechanisms and consequences of pan-membrane pyroptosis, our study advances the understanding of GSDM biology and its therapeutic potential. These findings not only highlight the risks of uncontrolled pyroptosis but also provide actionable insights for developing safer, more effective GSDM-based therapies. As the field moves toward clinical translation, a balanced approach that maximizes therapeutic efficacy while minimizing systemic toxicity will be essential.

## MATERIALS AND METHODS

The research involving animal studies described in this work adhered to strict ethical guidelines as established by the Chinese Regulations of Laboratory Animals, and Laboratory Animal—Requirements of Environment and Housing Facilities. Prior to conducting the animal experiments, all animal experiment protocols were reviewed and approved by the Institutional Animal Care and Use Committee of the Academy of Military Medical Sciences (Permit number: IACUC-DWZX-2024-011). The researchers involved in the study took all necessary measures to minimize animal suffering and discomfort. Careful attention was given to the housing conditions, handling and overall well-being of the animals throughout the duration of the study.

Detailed descriptions of materials and methods are available as Supplementary data at *NSR* online.

## Supplementary Material

nwaf452_Supplemental_Files

## Data Availability

All data are available in the main text or from the authors upon request.
