# Supplementary material for: Pan-membrane pyroptosis of liver induced by gasdermin-encoding mRNAs
Source: Natl Sci Rev. 2025 Oct 21;13(3):nwaf452. doi: 10.1093/nsr/nwaf452 (PMC12875121; doi:10.1093/nsr/nwaf452)
Supplement: nwaf452_Supplemental_Files [file nwaf452_supplemental_files.zip › Final-S-In vivo pyroptosis-HYJ-251017.docx]

**Supplementary methods**

***Ethics statement***

The research involving animal studies described in this work adhered to strict ethical guidelines as established by the Chinese Regulations of Laboratory Animals and Laboratory Animal-Requirements of Environment and Housing Facilities. Prior to conducting the animal experiments, all animal experiment protocols were reviewed and approved by Institutional Animal Care and Use Committee of Academy of Military Medical Sciences (Permit number: IACUC-DWZX-2024-011). The researchers involved in the study took all necessary measures to minimize animal suffering and discomfort. Careful attention was given to the housing conditions, handling, and overall well-being of the animals throughout the duration of the study.

***Cell culture***

Human cervical cancer cell line HeLa (ATCC, CCL-2) and human embryonic kidney cell line HEK293T (ATCC, CRL-11268) were cultured in Dulbecco's Modified Eagle's Medium (DMEM) (Gibco, 11995065) supplemented with 10% fetal bovine serum (Gibco, A5670701) and 100 U/ml penicillin and 100 µg/ml streptomycin (Gibco, 15140122) at 37°C in a humidified incubator with 5% CO_2_ humidified incubator. Mouse malignant melanoma cell line (B16-F10) (a gift from National Center of Biomedical Analysis (NCBA)) was cultured in RPMI 1640 medium (Gibco, 22400089) supplemented with 10% fetal bovine serum and 100 U/ml penicillin and 100 µg/ml streptomycin under the same conditions.

***Animals***

Seven-week-old female BALB/c and eight-week-old male C57BL/6J were purchased from Beijing Vital River Laboratory Animal Technology Co., Ltd.. Seven-week-old female immunodeficient B-NDG mice (on a BALB/c background) were purchased from Beijing Biocytogen Co., Ltd.. The choice of mouse sex was not specific, as either gender could be used for the experiments. Seven *Cynomolgus macaques*, all aged six, including three females and four males, were purchased from Guangzhou Xusheng Biotechnology Co., Ltd., and housed in a standard cleanliness animal facility. Animals were randomly assigned to the experimental and control groups.

***mRNA synthesis***

The mRNAs were synthesized in vitro using the T7 High Yield RNA Transcription Kit (Vazyme, TR101-01) with linearized DNA templates obtained from plasmids (GenScript). These plasmids were specifically designed to encode the N-terminal region of human Gasdermins and incorporate both 5’ and 3’ untranslated regions. Additionally, a poly-A tail longer than 100 adenine residues in length was designed at the end of the mRNA.

***Lipid-nanoparticle encapsulation of the mRNA***

Lipid-nanoparticle (LNP) formulations were prepared following the previously established procedure[1]. The lipid composition of LNPs used for targeting different organs may vary slightly. Here is a brief overview of LNP formulation specifically targeting the liver. Initially, lipids including 1,2-distearoyl-sn-glycero-3-phosphocholine (DSPC), cholesterol, and PEG-lipid were dissolved in ethanol at molar ratios of 50:10:38.5:1.5. The lipid mixture was then combined with 20 mM citrate buffer (pH 4.0, Teknova, Q2444) containing mRNA at a ratio of 1:2 using the NanoAssemblr Ignite^TM^ (Cytiva). Subsequently, the formulation was then dialyzed in PBS (pH 7.4, Gibco, 70011044) using a 20 kDa molecular weight cutoff dialysis tubing and ultrafiltered to achieve the desired concentration. The final product was filtered through a 0.22 µm filter and stored at 4°C. Particle size, distribution, RNA concentration, and encapsulation efficiency were assessed for all formulations.

***mRNA transfection and Fluorescence imaging***

HEK 293T and HeLa cells were seeded in 12-well plates at a density of 100,000 cells per well. After 12 hours, the cells were transfected with *GSDMD^NT^* mRNA (0.5 μg/well) using Lipofectamine 3000 Transfection Reagent (Thermo Fisher Scientiﬁc, L3000015). Six hours post-transfection, Annexin A5-FITC and PI (Vazyme, A211-01) were added to the medium. After incubating for 5 minutes, fluorescence imaging was carried out using the PerkinElmer Operetta CLS high content analysis system.

***Lactate dehydrogenase (LDH) release***

HEK293T cells were plated in 12-well plates at a concentration of 100,000 cells per well with 5% fetal bovine serum. After 12 hours, the cells were transfected with 400 ng/well of N-terminal gasdermins mRNAs using Lipofectamine 3000 Transfection Reagent (Thermo Fisher Scientific, L3000015). Cell death was evaluated using the CytoTox 96^®^ Non-Radioactive Cytotoxicity Assay Kit (Promega, G1780), following the protocol provided by the manufacturer. In brief, 50 µL of supernatant from each well was transferred to a 96-well plate. An equal volume of substrate mix was added, and the plates were incubated at room temperature for 30 minutes, protected from light. The reaction was stopped by adding 50 µL of stop solution, and absorbance was measured at 490 nm using a microplate reader.

***Treatment of B16-F10 Tumor-Bearing Mice***

B16-F10 cells were harvested when they reached 90% confluency using 0.05% trypsin-EDTA (Gibco, 25300054) and washed twice with phosphate-buffered saline (PBS, Gibco, 10010023). The cells were then resuspended in PBS at a concentration of 1×10^6^ cells/100 μL.

Eight-week-old male C57BL/6J mice were obtained from Beijing Vital River Laboratory Animal Technology Co., Ltd. and acclimated for one week before the experiments. The mice were briefly anesthetized with 2% isoflurane, and the right flank area was shaved and sterilized with 70% ethanol. Subsequently, A 100 μL suspension of 1×10^6^ B16-F10 cells was then injected subcutaneously into the right flank of each mouse using a 27-gauge needle.

Six days post-injection, tumor-bearing mice were treated by intratumoral injection of *GSDMD^NT^* mRNA LNP or placebo (equivalent LNPs without encapsulated mRNA) every three days (2 µg per mouse per dose). The mice were monitored daily, and tumor growth was measured every three days using a digital caliper. Tumor volume was calculated using the formula: Volume =0.52×length×width^2^. Nine days after the treatment, the mice were sacrificed, and tissues were ﬁxed with 4% formaldehyde for subsequent histology analysis. The study was conducted with blinding to reduce bias. For quantification of TUNEL-positive cells in mouse liver tissues, the numbers of TUNEL-positive and total cells in liver sections were automatically analyzed using Aipathwell, an AI-based image analysis software (Servicebio).

***Blood biochemistry, count, and cytokine analysis***

Six hours after injecting *GSDMD^NT^* mRNA LNP into the tail vein of seven-week-old female BALB/c mice, blood samples were collected. A blood routine test was immediately performed using the BC-5000vet Auto Hematology Analyzer (Mindray, Shenzhen, China) to analyze various blood components.

For cytokine analysis, the collected blood samples were allowed to coagulate at room temperature for 1 hour. Serum was obtained by centrifugation at 6000 g for 10 minutes. The concentrations of the 36 cytokines were measured using the 36-Plex Mouse ProcartaPlex Panel (Invitrogen, EPX360-26092-901) and the Luminex 200 instrument, with the procedure conducted by Shanghai Laizee Biotech Co., Ltd. according to the manufacturer's instructions.

For blood biochemistry analysis, serum was collected 20 hours post-injection. Biochemical indicators, including ALT and AST, were measured using an automatic biochemistry apparatus (Hitachi*3100*, HITACHl, Tokyo, Japan) corresponding assay kits conducted by Beijing Biocytogen Co., Ltd..

Similar procedures were employed for blood biochemistry and cytokine analysis in other experiments involving other gasdermin N-terminal mRNAs and inhibitors of GSDMD. Randomization and blinding were applied where applicable to minimize bias.

***Treatment protocol and inhibitor administration***

Seven-week-old female BALB/c mice were intravenously injected with 2 µg *GSDMD^NT^* mRNA-LNP. Disulfiram (MCE, HY-B0240) was administered intraperitoneally at a dose of 50 mg/kg, using a solvent mixture of 5% DMSO and 95% saline containing 20% SBE-β-CD (MCE, HY-17031). The disulfiram treatment schedule included pretreatment at 24 hours and 4 hours before the *GSDMD^NT^* mRNA-LNP injection, as well as post-treatment at 24 hours and 48 hours after the injection following the protocol from a previous study[2].

Additionally, two other inhibitors, triamcinolone (MCE, HY-B0328, 5 mg/kg) and hydrocortisone (MCE, HY-N0583, 5 mg/kg) were administered intraperitoneally using the same solvent mixture. These treatments were given 4 hours before the injection and as post-treatments at 24 and 48 hours following the injection, based on established protocols[3,4].

Blood samples were collected 6 hours post-injection for cytokine analysis, and the survival of the mice was monitored for up to 72 hours post-injection.

***Intravital microscopy imaging of mouse liver***

Intravital microscopy of the mouse liver was performed following an established abdominal window preparation protocol[5]. In this experiment, *Gt(ROSA)26Sor^tm4(ACTB-tdTomato,-EGFP)Luo^*/J (mT/mG, also known as ROSA mT/mG, Jax 007576) mice, which express the membrane-localized fluorescent protein mtdTomato, were utilized to visualize changes in the membrane structure of mouse hepatocytes. Following surgical creation of the abdominal window, *GSDMD^NT^* mRNA-LNP, FITC-labeled dextran (for blood vessel labelling), and Hoechst (for nuclear labelling) were injected into the tail vein. The mice were anesthetized and positioned under an inverted confocal microscope for in vivo cell imaging. Images were captured at regular intervals of three minutes using Nikon ECLIPSE Ti2 confocal laser scanning microscope system to observe real-time changes in the hepatocytes.

***Cynomolgus Macaque administration and sampling***

Seven Cynomolgus macaques were intravenously infused with GSDMDNT mRNA-LNP over a period of 0.5 to 1.5 hours at doses of 0.1 mpk, 0.25 mpk, or 1 mpk, respectively. For the 0.1 mpk group, blood samples were collected at specific time points post-injection (0, 6, 12, 24, and 72 hours) via venipuncture to assess liver function and cytokine levels. The concentrations of the 37 cytokines were measured using the 37-Plex NHP ProcartaPlex™ (Invitrogen, EPX370-40045-901) with the same procedures as mice. The Cynomolgus macaque in the 1 mpk group died approximately 6 hours post-injection, at which point they were immediately dissected for pathological examination of the liver tissue.

***Multiplex immunofluorescent assay***

Multiplex immunofluorescence staining was performed on paraffin sections. Briefly, 4-μm-thick sections were deparaffinized in xylene and rehydrated in a series of graded alcohols. Antigen retrievals were performed in citrate buffer (pH 6.0) by using a microwave oven for 20 minutes at 95 °C followed by a 20-minute cool down at room temperature. Multiplex fluorescence labelling was performed using TSA-dendron fluorophores (NEON 7-color IHC Kit for FFPE, Histova Biotechnology, NEFP7100). Briefly, primary antibody was incubated for 2 hours in a humidified chamber at 37 °C, followed by detection using the HRP conjugated secondary antibody and TSA-dendron fluorophores. Afterwards, the primary and secondary antibodies were thoroughly eliminated by heating the slides in retrieval/elution buffer (Abcracker 2in1 Retrieval/Fast Elution Solution, Histova Biotechnology, ABCFR5L) for 10 seconds at 95°C in a microwave. In a serial fashion, each antigen was labeled with distinct fluorophores. The multiplex antibody panels applied in this study were: cleaved-Gasdermin D (1:300, CST, #36425), cleaved-Caspase3 (1:400, CST, #9664), Glutamine synthetase (1:800, Abcam, ab176562), N-Cadherin (1:200, Abcam, ab207608), E-Cadherin (1:500, CST, #3195), liver Arginase (1:800, Abcam, ab233548), AIF (1:400, Abcam, ab32516), Calnexin (1:400, Abcam, ab92573), Cathepsin B (1:800, CST, #31718), Lamin A/B1/C (1:400, Abcam, ab108922), Tgoln1 (1:300, Abcam, ab283678), IL-1 beta (1:400, Abcam, ab283818), IL-18 (1:800, Abcam, ab223293; CST, #67775), CD31 (1:300, CST, #77699), NF-κB (1:400, CST, #8242), CDH5 (1:1000, Abcam, ab313632). After all the antibodies were detected sequentially, the slices were imaged using the confocal laser scanning microscopy platform Zeiss LSM880 equipped with AiryScan super-resolution module.

***Transmission electron microscopy (TEM) analysis***

For TEM sample preparation, chemical fixation and high-pressure freezing (HPF) methods were combined to and achieve optimal fixation. Briefly, mouse liver specimens were dissected into small pieces (less than 2 µm) and immediately immersed in glutaraldehyde (GA) at 4˚C for overnight fixation. Subsequently, the samples were thoroughly washed with sodium cacodylic acid buffer to remove residual GA before undergoing high-pressure freezing using the Leica HPF ICE system. Afterward, the specimens were treated with 2% OsO4 and subjected to freeze substitution at -80 ˚C for 24 hours, followed by gradual warming to room temperature. The removal of OsO4 was achieved through rinsing with acetone. The samples were then detached from planchettes and embedded in Embed-812 resin (Electron Microscopy Sciences, Cat. No. 14120)[6]. Thin sections (100 nm thick) were prepared from the sample blocks at each time point and placed on formvar-coated copper grids (Electron Microscopy Sciences). Staining was performed using 2% uranyl acetate (UA) in 70% methanol, followed by Reynold's lead citrate (LC)[7].Imaging was conducted using a Hitachi 7400 TEM (Hitachi-High Technologies) operating at 80 kV.

***Electron tomography (ET) and 3D modeling***

For dual-axis ET analysis, semi-thick sections (250 nm) were collected and stained following the aforementioned protocol. To facilitate dual-axis data alignment, 15 nm-gold particles were introduced to both sides of the section surface. Tilt series were acquired from 60° to -60° (with 1.5° intervals) using a Thermo Scientific™ Talos™ Arctica™ Cryo-TEM operating at 200 kV. Tomograms were reconstructed using the method described by Toyooka and Kang[8]. For model generation, the auto-contour command of the IMOD software package was employed, as previously described[9].

***Statistical analysis***

All data were analyzed using GraphPad Prism 8.4 software or custom Python scripts. Unless specified, data were presented as mean ± SEM in all animal experiments. Unpaired t test or two-way ANOVA were used to determine statistical significance among different groups (n.s., not significant; **P* < 0.05; ** *P* < 0.01; *** *P* < 0.001; **** *P* < 0.0001).

**Figure S1. GSDM-based therapy caused liver specific pathologic changes in mice.**

(A) HEK293T and HeLa cells were transfected with luciferase or *GSDMD^NT^*-encoding mRNA. Annexin A5-FITC and PI were added to the cell culture medium 12 hours post transfection to label the cell membrane and nucleus of dying cells.

(B) The intensity and mass distribution of *GSDMD^NT^* mRNA-LNP were measured using the Stunner from Unchained Labs. The light intensity profile shows a single prominent peak, indicating a monodisperse population with no significant aggregation, while the mass distribution confirms a predominant peak at approximately 72 nm.

(C) Schematic of the construction and treatment of B16-F10 tumor-bearing mice: A suspension of 1×10^6^ B16-F10 cells was subcutaneously injected into the right flank of eight-week-old male C57BL/6J mice (n=5). After 6 days, the mice were treated with *GSDMD^NT^* mRNA-LNP or placebo via intratumoral administration (i.t.) every 3 days. The mice were sacrificed 15 days after tumor cell transplantation.

(D to F) Tumor volume growth curves (D), weights (E) and sizes (F) of B16-F10 tumor-bearing mice treated with *GSDMD^NT^* mRNA-LNP or placebo. Tumor volume was calculated using the formula: Volume (mm^3^) = 0.52×length×width^2^.

(G) Serum levels of ALT and AST in B16-F10 tumor-bearing mice at 12 and 15 days after tumor cell transplantation.

(H and I) TUNEL staining (H) and H&E staining (I) of livers from B16-F10 tumor-bearing mice treated with *GSDMD^NT^* mRNA-LNP or placebo. The bar chart shows the ratio of TUNEL-positive cells to total cells in liver sections.

Boxed areas (I) in each image were shown in the middle at higher magnifications. Data in D, F, G and H are mean ± SEM, unpaired t test (n.s., not significant, **P* < 0.05, ** *P* < 0.01, *** *P* < 0.001, **** *P* < 0.0001).

**Figure S2. The Delivery of GSDMD^NT^ had no effect on blood routine but increased levels of inflammatory factors in mouse liver. Related to Figure 1.**

(A) Blood routine tests of mice injected i.v. with 5 μg of *GSDMD^NT^* mRNA-LNP or placebo for 6 hours (n=5).

(B) Oil red O staining of mouse livers 20 hours after tail vein injection of 5 μg *GSDMD^NT^* mRNA-LNP or placebo. Boxed areas in each image were shown in the middle at higher magnifications.

(C) Serum cytokine levels relative to Fig.1H. The histogram displays concentrations of cytokines. Data are shown as mean ± SEM, unpaired t test. (n.s., not significant; *P* < 0.05; ** *P* < 0.01; *** *P* < 0.001; **** *P* < 0.0001).

(D) Immunofluorescent analysis of inflammatory factors in mouse livers. Mice were treated with 5 µg *GSDMD^NT^* mRNA-LNP for 0, 12 or 20 h. CD31 (white) stained the blood vessel endothelial cells; DAPI (blue) stained the nuclei.

**Figure S3. GSDMD^NT^ targeted membranous organelles. Related to Figure 3.**

(A and B) Representative TEM images of mitochondria (A) or lysosomes (B) in hepatocytes of mice treated with 5 μg *GSDMD^NT^* mRNA-LNP or placebo.

(C) Immunofluorescent analysis of mouse hepatocytes showing nuclear envelops and lysosomal membranes. The mice were injected i.v. with 5 μg *GSDMD^NT^* mRNA-LNP or placebo for 3 hours. GSDMD^NT^ was stained in green (cleaved hGSDMD); nuclei were stained in blue (DAPI); nuclear envelops were stained in orange (Lamin A/B1/C); lysosomal membranes were stained in yellow (Cathepsin B).

(D) Time-lapse imaging of HEK293T cells over a period of 7 hours and 20 minutes. Lysosomes were labelled in green using Cell Light Lysosome-GFP, which was added to the cells 16 hours prior to the start of the experiment. Images were captured after *GSDMD^NT^* mRNA transfection. White arrows indicated changes of lysosomes; yellow arrows indicated outward bubbled plasma membranes. Nuclei were labelled in blue with Hoechst.

**Figure S4. The non-human primate was sensitive to GSDMD^NT^ treatment. Related to Figure 4.**

(A) Survival curves for cynomolgus macaques administered with *GSDMD^NT^* mRNA-LNP intravenously at doses of 0.1, 0.25 or 1 mpk.

(B) Immunofluorescent analysis of the liver from monkeys treated with 1 mpk *GSDMD^NT^* mRNA-LNP or placebo for approximately 6 h. CDH5 (yellow) stained the blood vessel endothelial cells.

**Figure S5. GSDMD^NT^’s pore-forming ability, rather than systemic immune responses, was the primary cause of mortality. Related to Figure 5.**

(A) LDH release in *GSDMD*^NT^–transfected HEK293T cells pretreated with disulfiram for 0.5 h.

(B) LDH release of HEK293T cells transfected with *Luciferase*, *GSDMD*^NT^, or *GSDMD*^NT^-*mut* mRNAs for 6 hours (n=3).

(C) Survival curves of mice intravenously injected with 5 μg *GSDMD*^NT^-*mut* mRNA-LNP or placebo (n=5). Statistical analysis of survival was performed with a log-rank (Mantel–Cox) test.

(E) Serum ALT and AST concentrations measured 20 hours after administration of 5 μg *GSDMD*^NT^-*mut* mRNA-LNP or placebo (n=5).

(E) H&E staining of mouse livers 20 hours after injection of 5 μg *GSDMD*^NT^-*mut* mRNA-LNP or placebo. Boxed areas are shown at higher magnifications. Scale bar, 20 μm.

(F) Survival curves of WT and B-NDG mice intravenously injected with 3 μg *GSDMD*^NT^ mRNA-LNP (n=5).

(G) Volcano plot of 36 cytokines in sera of B-NDG mice injected intravenously with 5 μg *GSDMD*^NT^ mRNA-LNP for 6 hours, relative to WT mice (n=5). Vertical dashed lines indicate a 1.5-fold increase or decrease.

Data in A, B, and D are shown as mean ± SEM, unpaired t test. (n.s., not significant; **** *P* < 0.0001).

**Figure S6. *GSDMs^NT^-Flag* mRNA induced pyroptosis *in vitro*. Related to Figure 6.**

(A) HEK293T cells were transfected with different *GSDMs^NT^-Flag* mRNAs respectively for 6 hours to assess LDH release, or for 2 hours to analyze the expression of GSDMs^NT^. β-actin was used as a loading control. Data are shown as mean ± SEM, unpaired t test (**** *P* < 0.0001).

**Figure S7. The schematic diagram of GSDMD^NT^ targeting blindly to plasma membranes and membranous organelles.**

A summary schematic diagram of the study. *GSDMD^NT^* mRNA-LNP was delivered into mice or non-human primates, triggered the damage of plasma membranes or intracellular membranous organelles, and ultimately led to cellular contents releasing, cytokine storms or liver dysfunction.

Supplementary references

1. Zhang NN, Li XF, Deng YQ, et al. A Thermostable mRNA Vaccine against COVID-19. *Cell*. Sep 3 2020;182(5):1271-1283 e16. doi:10.1016/j.cell.2020.07.024

2. Hu JJ, Liu X, Xia S, et al. FDA-approved disulfiram inhibits pyroptosis by blocking gasdermin D pore formation. *Nat Immunol*. Jul 2020;21(7):736-745. doi:10.1038/s41590-020-0669-6

3. Wu Z, Huang D, Xie J, Li M, Chen P, Yu Z. Triamcinolone acetonide suppressed scar formation in mice and human hypertrophic scar fibroblasts in a dose-dependent manner. *Cell Mol Biol (Noisy-le-grand)*. Aug 31 2023;69(8):226-231. doi:10.14715/cmb/2023.69.8.35

4. Jin L, Liao W, Zhou X, Wang Y, Qian J. Hydrocortisone alleviates sepsis-induced acute kidney injury through HSF-1-mediated transcriptional suppression of XPO1. *Tissue Cell*. Dec 2022;79:101915. doi:10.1016/j.tice.2022.101915

5. Wang Q, Liang Q, Dou J, et al. Breaking through the basement membrane barrier to improve nanotherapeutic delivery to tumours. *Nat Nanotechnol*. Jan 2024;19(1):95-105. doi:10.1038/s41565-023-01498-w

6. Kang BH. Electron microscopy and high-pressure freezing of Arabidopsis. *Methods Cell Biol*. 2010;96:259-83. doi:10.1016/S0091-679X(10)96012-3

7. Wang P, Chen X, Goldbeck C, Chung E, Kang BH. A distinct class of vesicles derived from the trans-Golgi mediates secretion of xylogalacturonan in the root border cell. *Plant J*. Nov 2017;92(4):596-610. doi:10.1111/tpj.13704

8. Toyooka K, Kang BH. Reconstructing plant cells in 3D by serial section electron tomography. *Methods Mol Biol*. 2014;1080:159-70. doi:10.1007/978-1-62703-643-6_13

9. Ma J, Liang Z, Zhao J, et al. Friendly mediates membrane depolarization-induced mitophagy in Arabidopsis. *Curr Biol*. May 10 2021;31(9):1931-1944 e4. doi:10.1016/j.cub.2021.02.034
