# Supplementary material for: Pan-membrane pyroptosis of liver induced by gasdermin-encoding mRNAs
Source: Natl Sci Rev. 2025 Oct 21;13(3):nwaf452. doi: 10.1093/nsr/nwaf452 (PMC12875121; doi:10.1093/nsr/nwaf452)

a

Blood test	Result (mean±SD)		Unit	Reference
	Placebo	*GSDMD*^NT mRNA-LNP		
WBC	2.73±0.33	3.91±0.85	10^9/L	0.8-10.6
Neu#	0.45±0.07	0.52±0.15	10^9/L	0.23-3.6
Lym#	2.22±0.28	3.32±0.98	10^9/L	0.6-8.9
Mon#	0.03±0.01	0.03±0.02	10^9/L	0.04-1.4
Eos#	0.03±0.01	0.03±0.01	10^9/L	0-0.51
Bas#	0.01±0.01	0.01±0.01	10^9/L	0-0.12
Neu%	16.32±2.19	14.82±8.75	%	6.5-50
Lym%	80.98±1.79	83.14±9.53	%	40-92
Mon%	1.06±0.49	0.94±0.76	%	0.9-18
Eos%	1.22±0.41	0.88±0.22	%	0-7.5
Bas%	0.42±0.4	0.22±0.04	%	0-1.5
RBC	9.48±0.31	9.28±0.35	10^12/L	6.5-11.5
HGB	165±8.25	158.8±4.92	g/L	110-165
HCT	43.26±1.49	42.92±1.43	%	35-55
MCV	45.64±0.56	46.24±0.25	fL	41-55
MCH	17.38±0.43	17.1±0.22	pg	13-18
MCHC	380.6±10.53	370.2±4.09	g/L	300-360
RDW-CV	14.96±0.15	14.1±0.32	%	45279
RDW-SD	31.44±0.67	29.66±0.67	fL	23-39
PLT	794.8±67.33	814.8±50.94	10^9/L	400-1600
MPV	5.52±0.22	5.44±0.15	fL	4-6.2
PDW	15.88±0.26	15.9±0.14		12-17.5
PCT	0.44±0.02	0.44±0.02	%	0.1-0.78

b

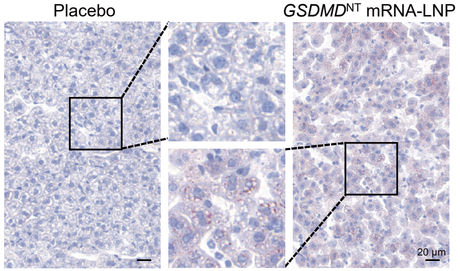

c

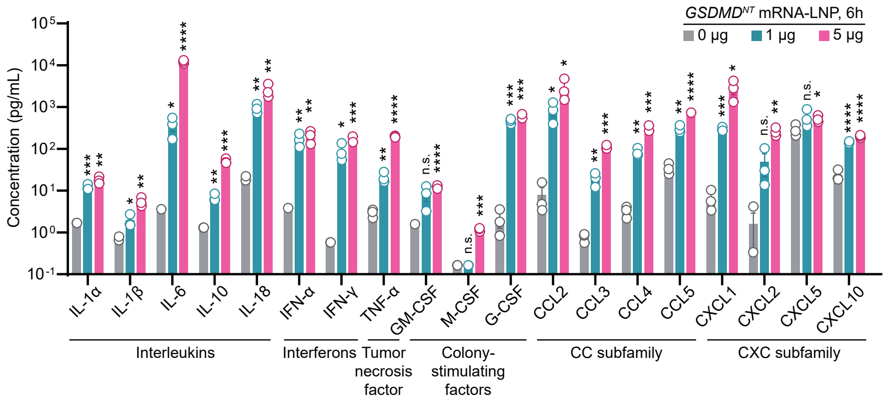

d

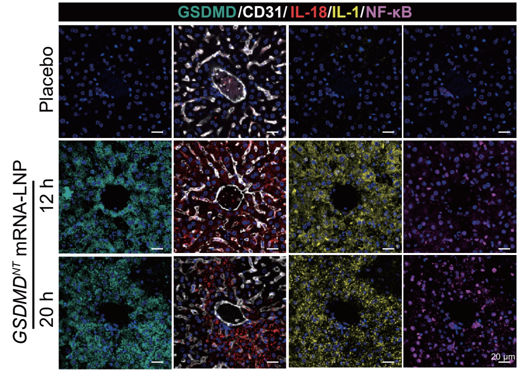

Supplement: nwaf452_Supplemental_Files [file nwaf452_supplemental_files.zip › S2.pdf]
