# Supplementary figures and images for: Pan-membrane pyroptosis of liver induced by gasdermin-encoding mRNAs
Source: Natl Sci Rev. 2025 Oct 21;13(3):nwaf452. doi: 10.1093/nsr/nwaf452 (PMC12875121; doi:10.1093/nsr/nwaf452)

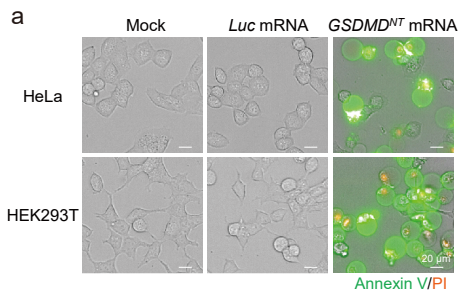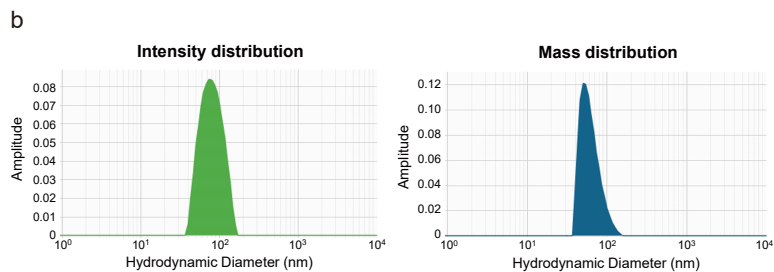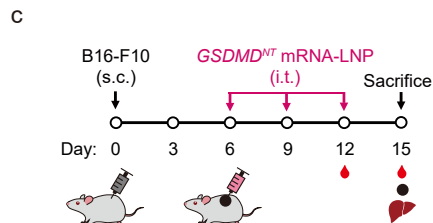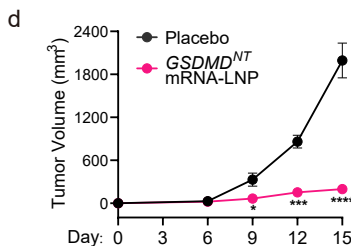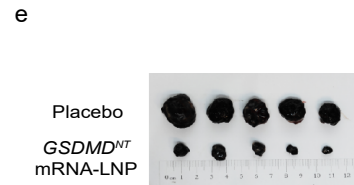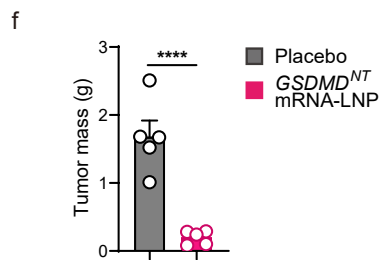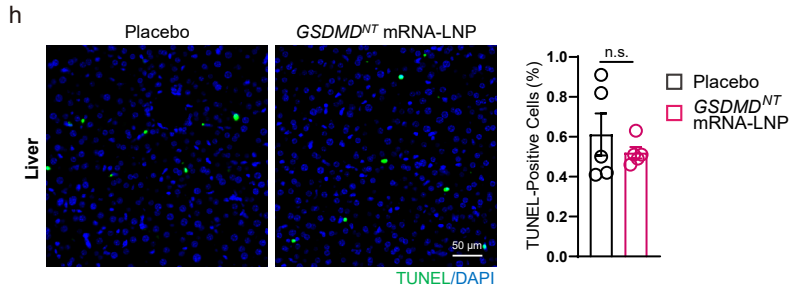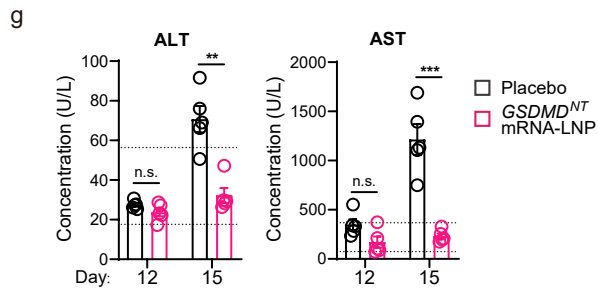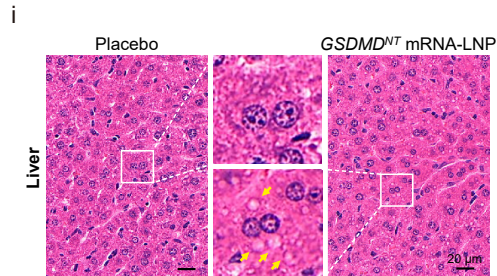

Supplement: nwaf452_Supplemental_Files [file nwaf452_supplemental_files.zip › S1.pdf]

a

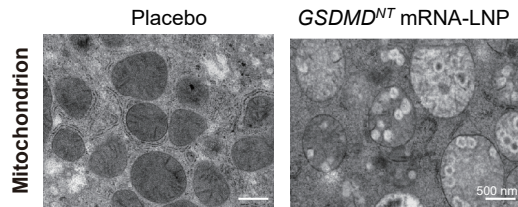

b

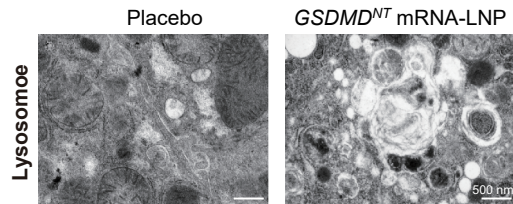

c

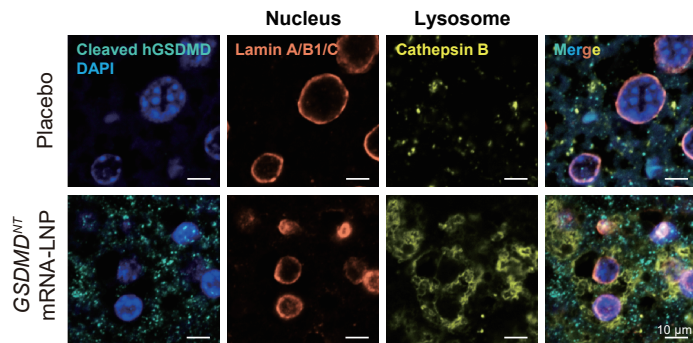

d

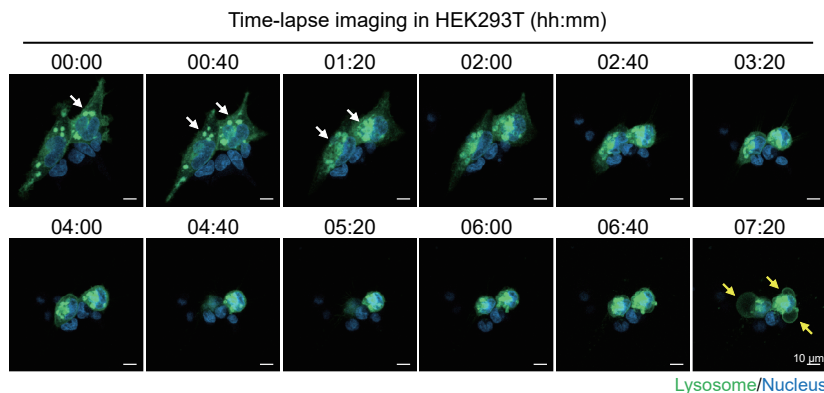

Supplement: nwaf452_Supplemental_Files [file nwaf452_supplemental_files.zip › S3.pdf]

a

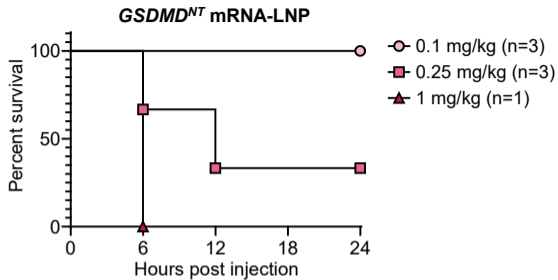

b

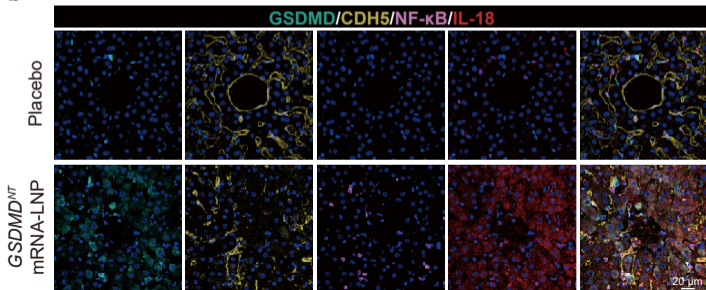

Supplement: nwaf452_Supplemental_Files [file nwaf452_supplemental_files.zip › S4.pdf]

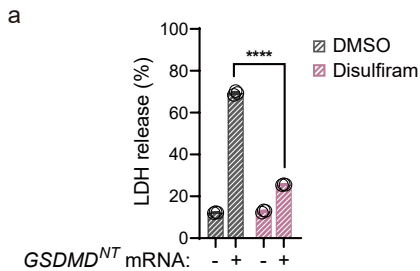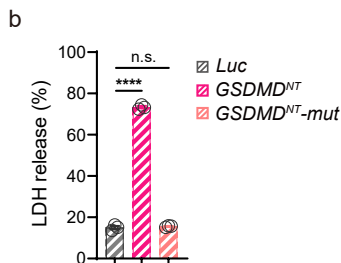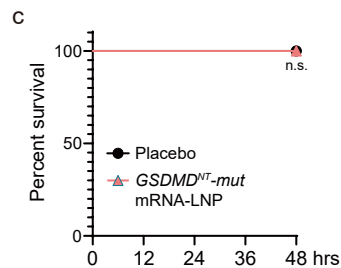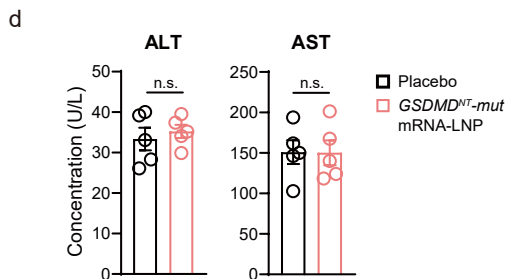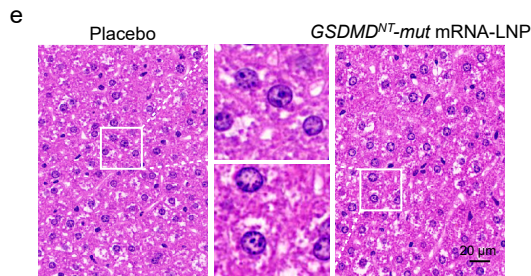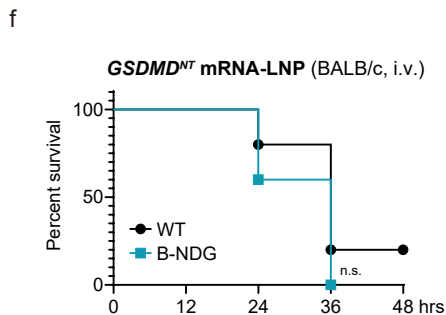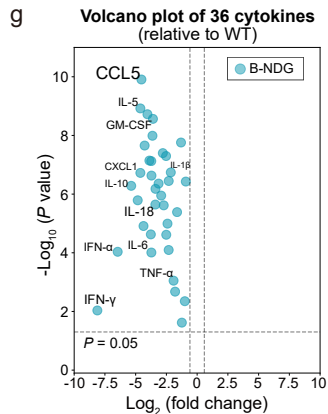

Supplement: nwaf452_Supplemental_Files [file nwaf452_supplemental_files.zip › S5.pdf]

a

*GSDMs<sup>NT</sup>-Flag mRNA*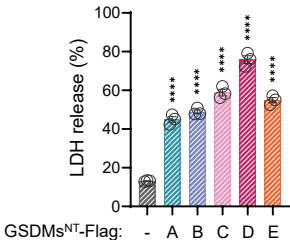

Flag

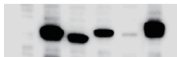 $\beta$ -actin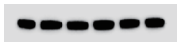

Supplement: nwaf452_Supplemental_Files [file nwaf452_supplemental_files.zip › S6.pdf]

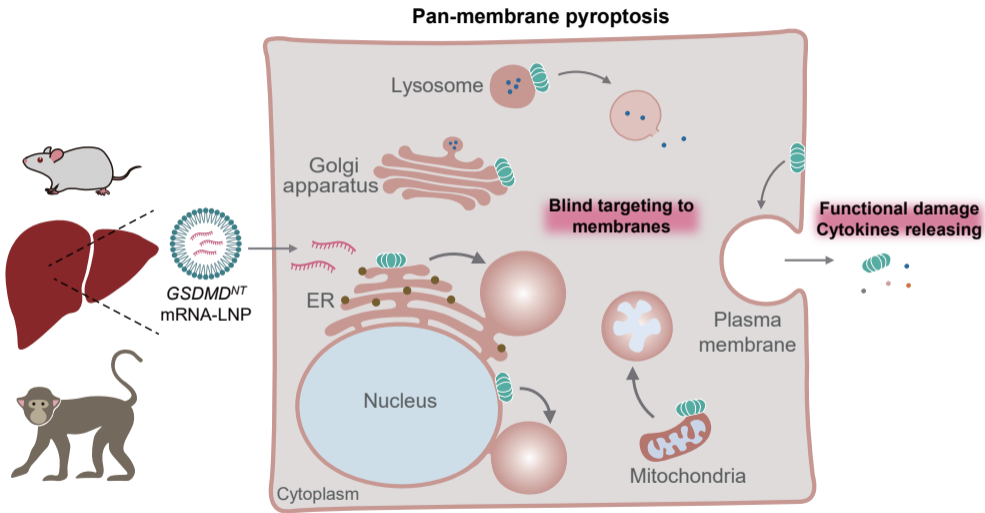

Supplement: nwaf452_Supplemental_Files [file nwaf452_supplemental_files.zip › S7.pdf]
